# Comparing random dot motion in MATLAB vs. Inquisit Millisecond

**DOI:** 10.3389/fpsyg.2022.1035518

**Published:** 2022-12-06

**Authors:** Kimia C. Yaghoubi, Sarah Kabbara, Sara Arian, Hadi Kobaissi, Megan A. K. Peters, Aaron R. Seitz

**Affiliations:** ^1^Perception and Learning Laboratory, Department of Psychology, University of California, Riverside, Riverside, CA, United States; ^2^Cognitive and Neural Computation Laboratory, Department of Cognitive Sciences, University of California, Irvine, Irvine, CA, United States; ^3^Center for the Neurobiology of Learning and Memory, University of California, Irvine, Irvine, CA, United States

**Keywords:** motion perception, random dot motion, direction estimation, intra-platform validity, inter-platform validity, COVID-19

## Abstract

Random Dot Motion (RDM) displays refer to clouds of independently moving dots that can be parametrically manipulated to provide a perception of the overall cloud moving coherently in a specified direction of motion. As a well-studied probe of motion perception, RDMs have been widely employed to understand underlying neural mechanisms of motion perception, perceptual decision-making, and perceptual learning, among other processes. Despite their wide use, RDM stimuli implementation is highly dependent on the parameters and the generation algorithm of the stimuli; both can greatly influence behavioral performance on RDM tasks. With the advent of the COVID pandemic and an increased need for more accessible platforms, we aimed to validate a novel RDM paradigm on Inquisit Millisecond, a platform for the online administration of cognitive and neuropsychological tests and assessments. We directly compared, in the same participants using the same display, a novel RDM paradigm on both Inquisit Millisecond and MATLAB with Psychtoolbox. We found that psychometric functions of Coherence largely match between Inquisit Millisecond and MATLAB, as do the effects of Duration. These data demonstrate that the Millisecond RDM provides data largely consistent with those previously found in laboratory-based systems, and the present findings can serve as a reference point for expected thresholds for when these procedures are used remotely on different platforms.

## Introduction

Here, we seek to validate a novel Random Dot Motion (RDM) procedure that can be broadly accessible for research and teaching purposes. RDM refers to clouds of independently moving dots that can be parametrically manipulated to induce a perception of the overall cloud moving coherently in a specified direction of motion. RDMs provide a useful probe of motion perception, given the ability to parametrically control the relative saliency of the motion stimuli. They have been employed in psychophysical and neurophysiological experiments to understand underlying neural mechanisms of motion perception (Britten et al., 1992; Purushothaman and Bradley, 2005), perceptual decision-making (Mazurek et al., 2003; Gold and Shadlen, 2007; Beck et al., 2008), perceptual learning (Ball and Sekuler, 1982; Zohary et al., 1994; Zanker, 1999; Seitz and Watanabe, 2003; Seitz et al., 2005; Law and Gold, 2008), motion direction discrimination (Adelson and Bergen, 1985; Cleary and Braddick, 1990; Britten et al., 1996; Banton et al., 2001; Pavan et al., 2016), depth perception (Kim and Mollon, 2002; Nadler et al., 2008; Kim et al., 2016), and short-term visual memory (Pavan et al., 2013, 2021) among other processes. While RDMs have emerged as one of the most conventional psychophysical paradigms for studying properties of visual motion perception processes, to date, RDM-based tasks are largely regulated to specialize in laboratory-based software systems (e.g., Psychophysics Toolbox for MATLAB, PsychoPy, Eprime, etc.) that are often associated with a steep learning curve, which prevents broad adaptation to students, younger researchers, clinicians, and others in early learning phases.

A challenge regarding the development of an accessible RDM-based research program is that the stimuli are somewhat complicated to generate. RDM stimuli consist of several frames in which a set of dots (often 50–200 or more) moves within an aperture, where the displacement of each dot must be calculated independently frame by frame. Further, to control for spatial cues that might indicate the direction of the dot-cloud then, when a dot exits the aperture then, a new dot needs to be generated on the opposite side of the aperture. To display such a sequence, not only does one need to generate the trajectories of the dot clouds, but one must also display them at an appropriate frame rate so that the user perceives the stimulus as intended. A further challenge is that most tasks using RDMs require these dot clouds to be generated in real-time and often in response to the performance of the participant. For instance, to estimate the RDM Coherence threshold (e.g., how well someone can perceive the motion of a small number of coherently moving dots in a cloud of otherwise randomly moving dots), it is typical for the direction, speed, and level of Coherence (e.g., the percent of dots moving in the targeted direction frame-to-frame) to change across trials, requiring a unique RDM stimulus to be generated in correspondence with adaptive procedures employed. In other words, it is not a trivial task to accurately generate or display such RDM stimuli.

However, as accentuated by the COVID pandemic, there is an increasing need for accessible platforms that can make and deploy RDM tasks, as well as other procedures, more broadly accessible and easy to use. Whether for teaching, research, or clinical use, it is important that these alternative systems can generate stimuli that have known psychometric properties. This is a particular challenge for RDMs, where the perception of RDM stimuli can be highly dependent upon both the parameters (speed, density, aperture, duration, frame rate, visual angles of dot-displacement, etc.) and the generation algorithm of the stimuli (e.g., are randomly moving dots displaced to random new locations or do they move with a fixed speed but random direction, do dots have limited lifetimes, do the same dots move coherently frame by frame, etc.) with several studies systematically showing that precise implementation can influence behavioral performance (Watamaniuk and Sekuler, 1992; Benton and Curran, 2009; Pilly and Seitz, 2009).

To this end, we collaborated with Millisecond, a leading provider of software platforms for the online administration of cognitive and neuropsychological tests and assessments. Inquisit Millisecond has been used in many peer-reviewed publications on a diverse range of topics, including but not limited to cognitive neuroscience, neuropsychology, clinical trials, marketing, human factors, and behavioral economics (Hillman et al., 2004; Van Damme et al., 2004, 2007; Smith et al., 2005; Vogt et al., 2008; Zakrzewska and Brzezicka, 2014; Bianchi and Laurent, 2015; Stewart et al., 2017; Jin and Lin, 2022; Smolker et al., 2022). While hundreds of experiments are programmed and tested in the Millisecond Library, until now, the RDM paradigm has not been available. In this study, we co-designed a novel RDM paradigm with Millisecond that they implemented in their Inquisit software package to closely match conventional RDMs used in MATLAB, which is implemented via PsychToolbox (Watamaniuk and Sekuler, 1992; Benton and Curran, 2009; Pilly and Seitz, 2009). The advantage of this task is that it is broadly accessible, cross-platform, and can be run on both participants’ own computers and in specialized laboratory environments. Further, anyone can try out tasks for free in the Millisecond library, which makes the procedure accessible for teaching (although there are fees associated with laboratory or clinical use of the platform).

In the current study, we directly compare, in the same participants using the same display, a MATLAB-based RDM [the dotsX.m function written by Seitz and used in (Pilly and Seitz, 2009)], implemented *via* the Psychophysics Toolbox extensions version 3 (Brainard, 1997; Pelli, 1997; Kleiner et al., 2007) compared to the novel Millisecond implementation. We find that psychometric functions of coherence largely match between the platforms, as are the effects of Duration. Further, we show that both programs have high inter-test reliability as well as high intra-test reliability between the platforms. These data demonstrate that the Millisecond RDM provides data largely consistent with those previously found in laboratory-based systems, and the present data can serve as a reference point for expected thresholds for when these procedures are used remotely on different platforms.

## Materials and methods

### Participants

Forty-four participants (ages 18–30) were recruited through the University of California, Riverside Psychology Research Participation System (SONA Systems) and were compensated for their research participation by course research credit. We had targeted 50 participants as this is the standard sample size that we have used for validation studies in our lab, and recruitment was cut-off at the end of the academic quarter. All participants had normal or corrected-to-normal vision and were naive in performing the task. All participants provided informed written consent as approved by the University of California, Riverside Institutional Review Board and in accordance with the Declaration of Helsinki.

### Apparatus

Participants sat on an adjustable height chair at a distance of 60 cm from a 36 cm horizontally wide ViewSonic PF817 CRT monitor set to a resolution of 1920 × 1440 and a refresh rate of 85 Hz. The distance between the participant’s eyes and the monitor was fixed by having them position their head on a chin-rest. The experiment was set up such that participants’ eyes and the monitor center were at the same horizontal level. Stimuli were presented *via* MATLAB, using the Psychophysics Toolbox extensions version 3 (Brainard, 1997; Pelli, 1997; Kleiner et al., 2007) and Millisecond Inquisit Lab version 6.5.2 (Millisecond Software, LLC) using a 2015 edition Apple MacBook Pro running OS Big Sur version 11.2.3.

### Stimuli

Motion stimuli consisted of RDM displays: white dots moving at a speed of 9.2 °/s on a gray background. Each dot was a 2 × 2-pixel square. A total of 100 dots were presented on every frame. Dots were displayed within an 11° diameter circular aperture that had no marked edges and was centered on the screen. Dot density was fixed across frames and trials at 16.7 dots per visual deg^−2^ S^−1^ (Pilly and Seitz, 2009). Every dot was generated to have a three-frame lifetime; at each frame transition, a set of dots moved in a coherent direction with the same speed while the rest of the dots were randomly repositioned within the aperture.

### Experimental procedure

**Figure 1:**
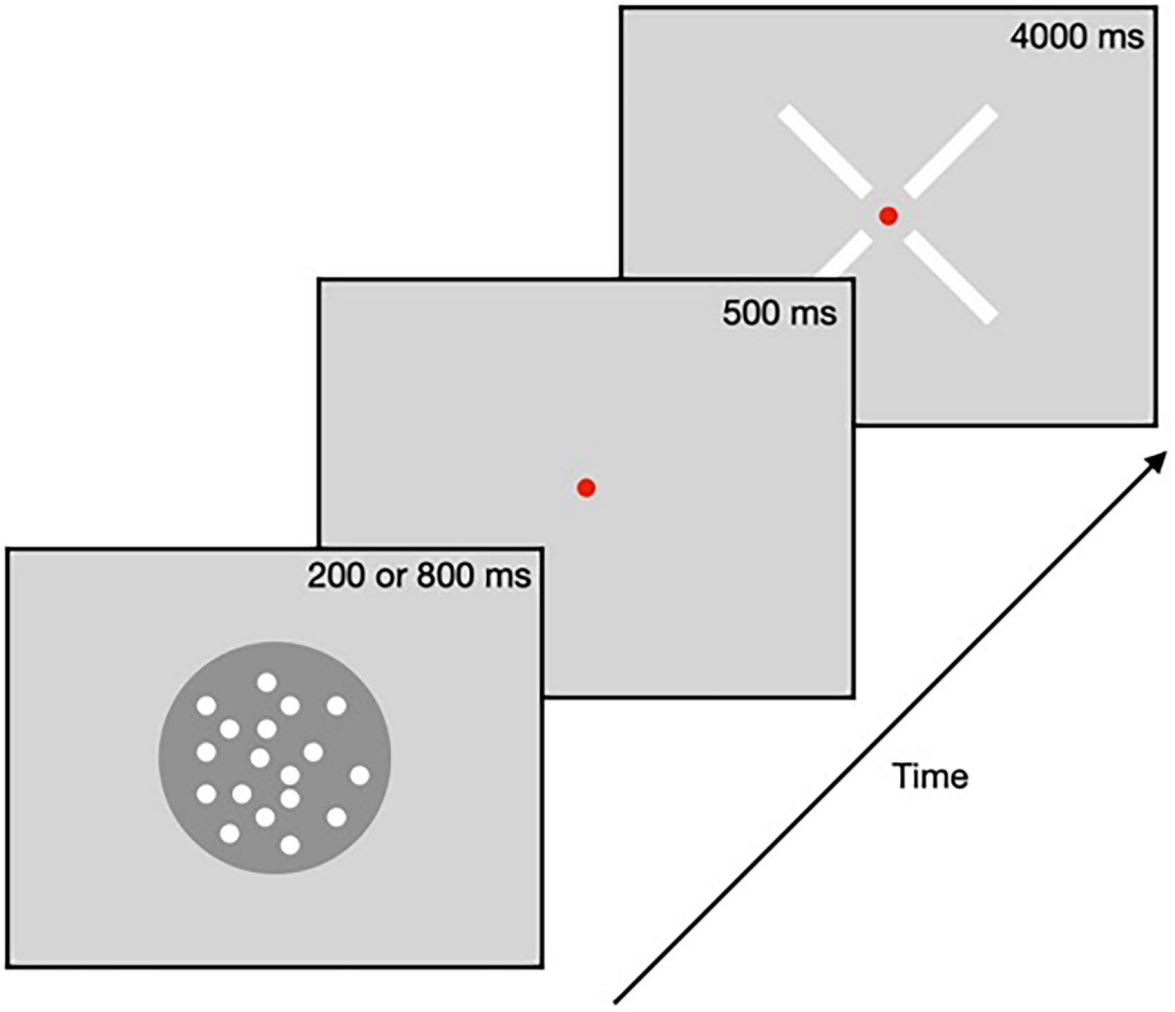
Task procedure. Random dot motion stimuli were presented for one of two Durations (either 200 ms or 800 ms) followed by a delay period of 500 ms, and then a response cross appeared, which participants clicked to register the perceived direction of motion.

Parameters closely followed the experimental paradigm implemented by Pilly and Seitz (2009). The experiment was conducted in a dark experimental room. Participants were instructed to fixate a 0.2° red fixation point presented at the center of the screen and to avoid tracking any particular dots during the stimulus presentation. In each trial, participants were presented with the RDM for a Duration of either 200 or 800 ms and then had 4 s to report the perceived direction of motion by clicking on one of four bars that corresponded to the possible directions of motion (Figure 1). If participants’ response was within 22.5° of the presented direction, then participants received visual feedback that their response was correct. For every trial, participants were instructed to fixate on the central spot.

All participants completed two experimental sessions, one for each Platform, in counterbalanced order. In each session, participants first completed a practice block consisting of 10 trials to familiarize themselves with the procedure. The method of constant stimuli was employed, such that the random dot motion was presented at two fixed Durations (200 or 800 ms) at 10 different Coherence levels (2, 4, 6, 8, 10, 15, 20, 25, 30, and 50%) and at one of four directions (45, 135, 225, and 315 degrees). Each session was comprised of 640 trials that were divided into four task blocks, with a self-paced break between each block. The RDM paradigm used in this study is available online on Inquisit Millisecond’s library.^1^

### Data analysis

Percent correct as a function of coherence is calculated as the total number of correct trials (per coherence level) divided by the total number of trials (per coherence level). Based on this an accuracy value between 0 and 1 is obtained and multiplied by 100 for reporting percent correct. To characterize performance, a three-way repeated measures ANOVA was run using the statistical platform JASP (JASP Team (2022). JASP (Version 0.16.3) [Computer software]), with Coherence, Duration, and Platform as the repeated measures factors, and the order of platform as the between subject factors. We used the same statistical software, JASP, to conduct Pearson’s correlation analysis between the average performance score for each subject on MATLAB versus Milliseconds.

## Results

**Figure 2:**
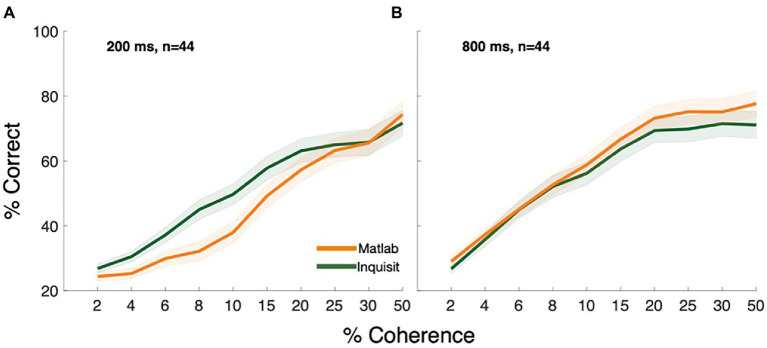
Coherence response functions of MATLAB and Inquisit Millisecond at viewing Durations **(A)** 200 ms and **(B)** 800 ms. Error is plotted as the standard error of the mean across all participants.

Overall, both MATLAB and Inquisit Millisecond Platforms led to well-characterized psychometric functions that were quite similar between the Platforms (Figure 2, RTs can be found in Supplementary Figure S1). To characterize performance, we performed a three-way repeated-measures ANOVA that evaluated the effects of Coherence, Platform (Inquisit or MATLAB), and Duration on participants’ behavioral performance. As expected, we found a significant effect of Coherence [*F*(9, 378) =139.0, *p* < 0.001, η_p_^2^ = 0.768], and Duration [*F*(1,42) =78.0, *p* < 0.001, η_p_^2^ = 0.651]. Importantly, we found no significant effect of Platform [*F*(1, 42) =0.755, *p* < 0.390, η_p_^2^ = 0.018]. However, there was a significant interaction between Coherence and Platform [F(9, 378) =6.0, *p* < 0.001, η_p_^2^ = 0.125] and between Coherence and Duration [*F*(9,378) = 10.4, p < 0.001, η_p_^2^ = 0.197]. Post-hoc comparisons can be found in Supplementary Tables S1, S2 in the Supplementary Figures. Overall, these results show that participants’ performance increases as a function of Coherence (η_p_^2^ = 0.768) and Duration (η_p_^2^ = 0.651), and that these relationships are not significantly affected by the Platform (η_p_^2^ = 0.125). These data suggest that while there are some differences between the psychometric functions – for example, a slight advantage for Inquisit at the brief viewing Duration – there is substantial similarity in performance across the two platforms.

**Figure 3:**
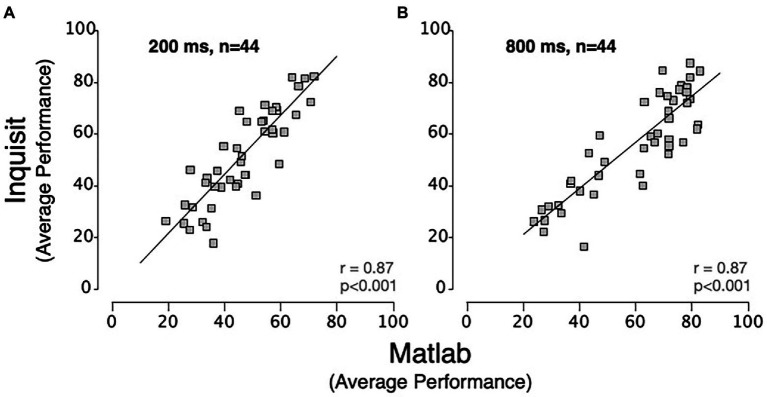
Scatter plots showing inter-platform relationships between MATLAB and Inquisit Millisecond for **(A)** 200 ms and **(B)** 800 ms RDM Durations. Each data point represents the mean performance of a single participant for each platform. The lines are trendlines fitted to the data (Pearson’s correlation: *p* < 0.001 for both 200 ms and 800 ms duration).

**Figure 4:**
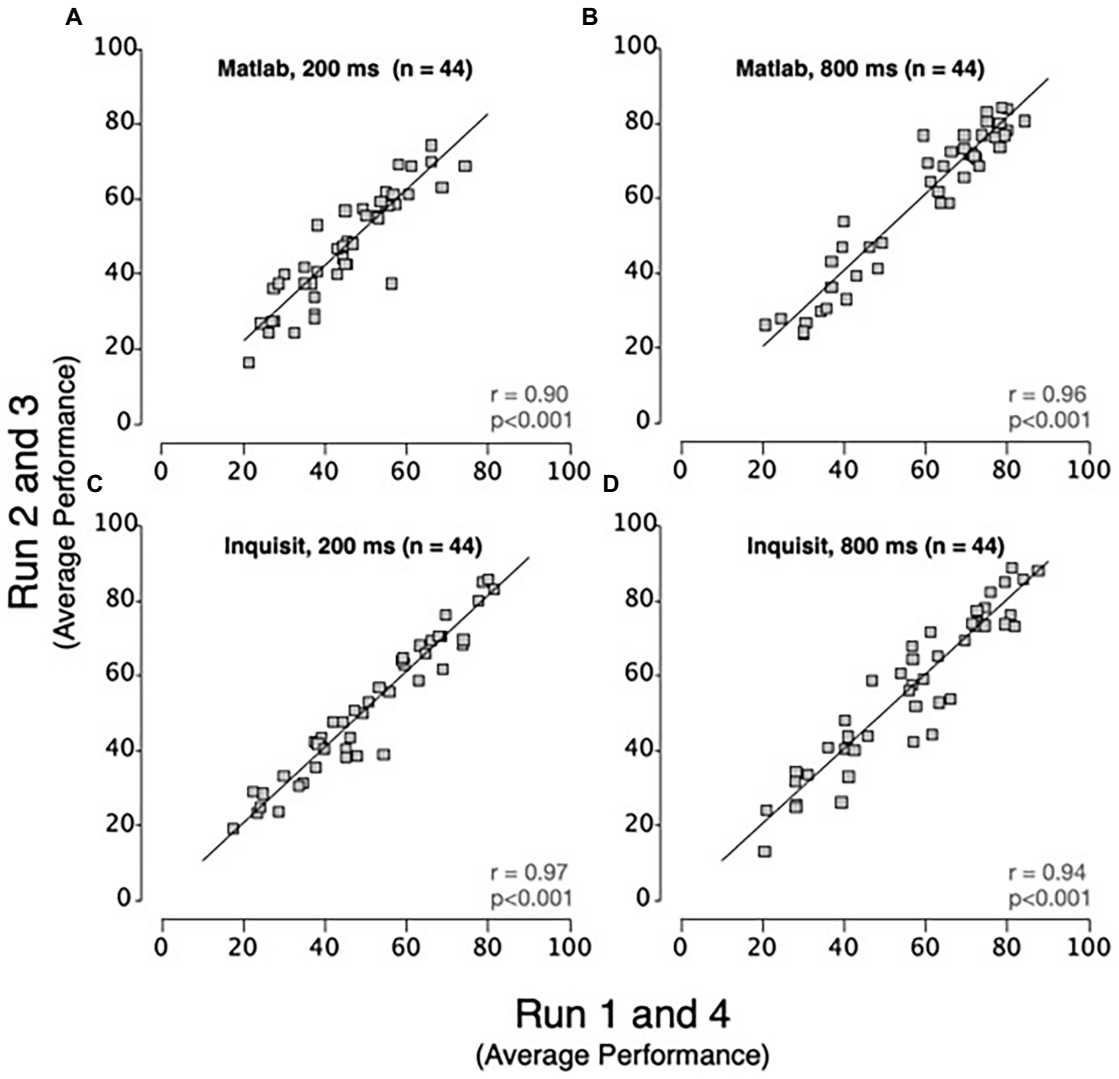
Scatter plots showing intra-platform relationships for MATLAB and Inquisit Millisecond platforms at 200 ms and 800 ms RDM durations. Each data point represents the mean performance of a single participant for runs 1 and 4 vs. runs 2 and 3 on MATLAB at **(A)** 200 ms and **(B)** 800 ms and on Inquisit Millisecond at **(C)** 200 ms and **(D)** 800 ms. The line is a trendline fitted to the data (Pearson’s correlation: *p* < 0.001 for both 200 ms and 800 ms durations on MATLAB and Inquisit Millisecond).

A key consideration in evaluating the relationship between the platforms is the extent to which they are evaluating the same construct. To address this, we examined inter-platform reliability. This was addressed by correlating the mean of each participant’s performance between platforms (Figure 3). We found that there were high inter-platform correlations for both viewing Durations (200 ms, *r* = 0.866, *p* < 0.001 and 800 ms, *r* = 0.870, *p* < 0.001). The significant inter-platform reliability shows that the Inquisit Millisecond platform leads to measurements that are largely consistent with RDM displays generated by MATLAB with Psychtoolbox. For context, we also examined intra-test correlations. To estimate these, we averaged performance accuracy across the first and fourth experimental runs and analyzed its correlation with the average of the second and third experimental runs. These intra-platform correlations (Figure 4) were also quite high for MATLAB with Psychtoolbox (200 ms, *r* = 0.90, *p* < 0.001 and 800 ms, *r* = 0.96, *p* < 0.001) and Inquisit (200 ms, *r* = 0.97, *p* < 0.001, and 800 ms, *r* = 0.94, *p* < 0.001). We note that while intra-platform correlations are numerically greater than inter-platform correlations, the intra-platform correlations are across sessions, while inter-platform correlations are within sessions. Overall, these data suggest that both platforms produce reliable estimates of motion perception, and that inter-platform reliability is reasonably comparable to intra-platform reliability.

## Discussion

The main contribution of this work is the validation of a cross-platform novel RDM method that can be run on participants’ own devices without the need for MATLAB and Psychtoolbox and is easily accessible to researchers, students, and clinicians. To validate a generic RDM-based task on the Inquisit Millisecond platform, in this study, we directly compared implementation and results from the same RDM task in MATLAB *via* Psychtoolbox and Inquisit Millisecond. Results show that psychometric functions of performance as a function of coherence are largely the same between MATLAB with Psychtoolbox and Inquisit Millisecond. Further, we found robust intra-platform and cross-platform reliabilities. These findings demonstrate that Millisecond RDM psychometric properties are consistent with MATLAB/Psychtoolbox and that the Millisecond RDM can provide high-quality and reliable data.

We note that the creation and presentation of RDM stimuli typically require technical sophistication and that the contribution of an accessible RDM that can be run remotely is particularly valuable given the increased focus on remote data collection that has been spurred by the COVID pandemic. While other platforms, including Psychophysics Toolbox for MATLAB and PsychoPy, can also support remotely-administered studies on participants’ own devices, these platforms require greater technical skills to use than the Millisecond Platform (and, in the case of MATLAB/Psychtoolbox, may involve installing commercial software that is not free). Given prior results showing that performance on RDM tasks is highly sensitive to the exact parameters used (Pilly and Seitz, 2009), it is particularly valuable to have an accessible RDM stimulus generator that has known psychometric properties.

However, we also note that there are some important limitations to the Millisecond RDM stimuli. The first issue is that while we compared performance between MATLAB and Millisecond on the same computer and the same monitor, the extent to which the Millisecond RDM performs consistently across different platforms remains to be clarified, as displays cannot be as easily controlled when using participant-owned devices. Of note, we did not address the extent to which frames may have been dropped or the extent to which this may be different between the platforms when run on different displays. However, we note that this is a limitation of any RDM that would be run on a participant’s device. We also note that while generally, in vision science, parameters are coded as degrees per visual angle, in Millisecond Inquisit, parameters are coded as percent screen size. However, given that visual angle is dependent upon the distance of the participant from the screen and percent screen size is dependent on the screen size, both Inquisit Millisecond and MATLAB with Psychtoolbox have limitations in uncontrolled settings, and both can easily be matched when the screen size is known. Additionally, online remedies such as virtual chinrests (Li et al., 2020) can be employed to minimize the change in stimulus perception. We note that for studies dependent upon a single measurement, these issues can be a significant concern; however, studies involving within-person designs, such as interventions or repeated testing, may be less impacted by these limitations.

## Conclusion

Overall results support using Millisecond RDM as a reliable method for characterizing motion discrimination performance in healthy human subjects. While further research will be required to understand the extent to which the Millisecond RDM is reliable when run at home on participant’s own devices, the present research sets the stage for this next step in validation. We do note that for any remotely-administered study, whether using Millisecond or another platform, careful design of the experimental parameters and cross-checking units may be required to ensure that data will be comparable to previously-reported results. Still, even with these limitations, this new RDM stimulus creation and deployment software can be of benefit to researchers, students, and clinicians as a reliable method to understand human motion processes.

## Data availability statement

The raw data supporting the conclusions of this article will be made available by the authors upon request, without undue reservation.

## Ethics statement

The studies involving human participants were reviewed and approved by University of California, Riverside (UCR) Institutional Review Board. The patients/participants provided their written informed consent to participate in this study.

## Author contributions

RDM algorithm in MATLAB was written by AS. Millisecond Inquisit RDM was written by Katja Borchert (Senior Consultant at Millisecond Software LLC) in consultation with AS and KY. Data were collected by undergraduate students SK, SA, and HK. Data analyses were conducted by KY. All authors assisted in the writing of the manuscript. All authors contributed to the article and approved the submitted version.

## Funding

This work was partially supported by a Canadian Institute for Advanced Research Azrieli Global Scholars Fellowship (to MP). The funders had no role in the study design, analysis, or conclusion.

## Conflict of interest

The authors declare that the research was conducted in the absence of any commercial or financial relationships that could be construed as a potential conflict of interest.

## Publisher’s note

All claims expressed in this article are solely those of the authors and do not necessarily represent those of their affiliated organizations, or those of the publisher, the editors and the reviewers. Any product that may be evaluated in this article, or claim that may be made by its manufacturer, is not guaranteed or endorsed by the publisher.

## Supplementary material

The Supplementary material for this article can be found online at: https://www.frontiersin.org/articles/10.3389/fpsyg.2022.1035518/full#supplementary-material

## Footnotes

^1^ https://www.millisecond.com/download/library/rdk
