# Supplementary material for: Comparing random dot motion in MATLAB vs. Inquisit Millisecond
Source: Front Psychol. 2022 Dec 6;13:1035518. doi: 10.3389/fpsyg.2022.1035518 (PMC9763265; doi:10.3389/fpsyg.2022.1035518)
Supplement: Supplementary file 1 [file Data_Sheet_1.PDF]

Supplementary Figures

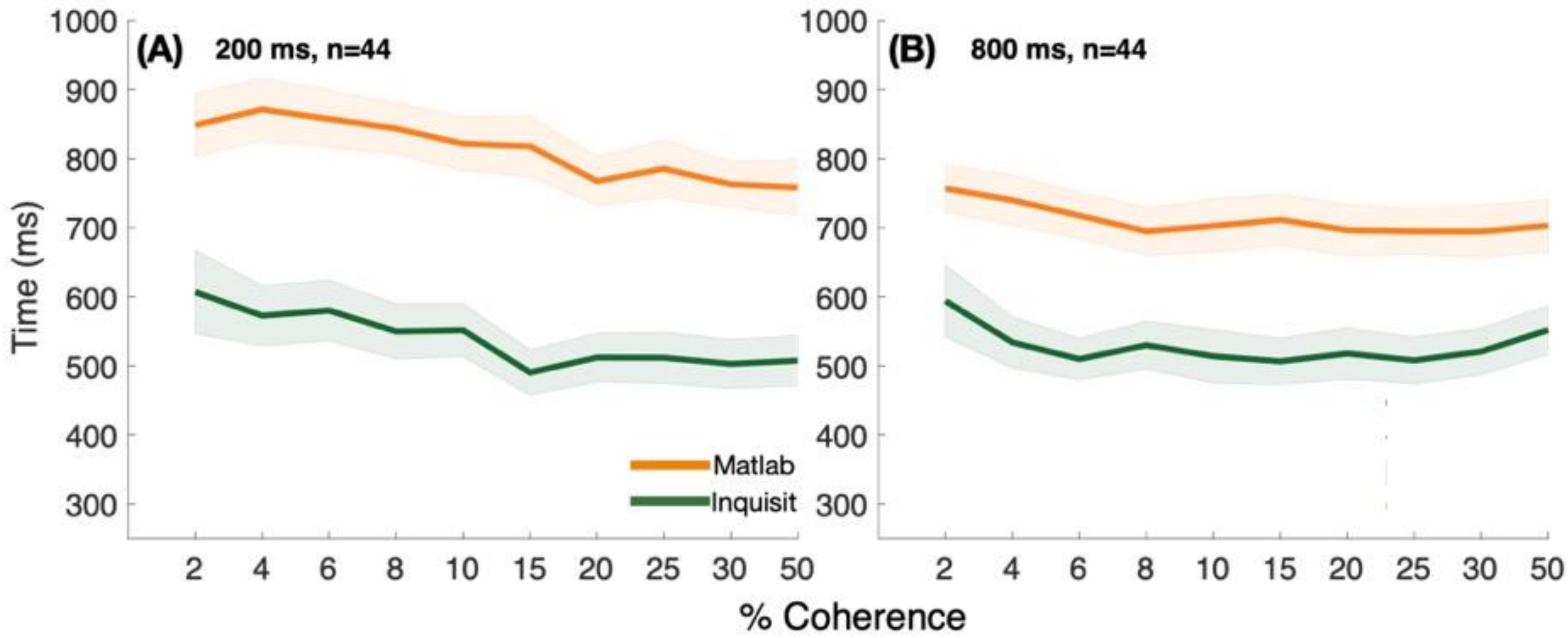

Figure S1. Reaction-time median for all correct trials as a function of coherence for the 200 ms duration (A) and 800 ms duration (B). Reaction-times difference between MATLAB and Inquisit Millisecond is due to a 500 ms delay post stimulus presentation that was present in Inquisit but was not implemented in MATLAB's RDM paradigm.

Post Hoc Tests

Post Hoc Comparisons - Platform ✻ Coherence								
		Mean Difference	SE	t	p_{tukey}	$p_{scheffe}$	p_{bonf}	p_{holm}
Millisecond, Coh 02%	Matlab, Coh 02%	0.079	1.715	0.046	1.000	1.000	1.000	1.000
Millisecond, Coh 04%	Matlab, Coh 04%	1.736	1.715	1.012	1.000	1.000	1.000	1.000
Millisecond, Coh 06%	Matlab, Coh 06%	3.457	1.715	2.015	0.892	1.000	1.000	1.000
Millisecond, Coh 08%	Matlab, Coh 08%	5.906	1.715	3.443	0.079	0.885	0.137	0.036
Millisecond, Coh 10%	Matlab, Coh 10%	4.322	1.715	2.520	0.570	0.996	1.000	0.442
Millisecond, Coh 15%	Matlab, Coh 15%	2.500	1.715	1.458	0.996	1.000	1.000	1.000
Millisecond, Coh 20%	Matlab, Coh 20%	0.801	1.715	0.467	1.000	1.000	1.000	1.000
Millisecond, Coh 25%	Matlab, Coh 25%	-1.984	1.715	-1.157	1.000	1.000	1.000	1.000
Millisecond, Coh 30%	Matlab, Coh 30%	-1.967	1.715	-1.147	1.000	1.000	1.000	1.000
Millisecond, Coh 50%	Matlab, Coh 50%	-4.778	1.715	-2.786	0.375	0.987	1.000	0.237
Note. **P-value adjusted for comparing a family of 190**								
Note. **Results are averaged over the levels of: order, Dur**								

Table S1. Post Hoc Comparisons - Platform ✻ Coherence.

Post Hoc Tests

Post Hoc Comparisons - Duration ✻ Coherence								
		Mean Difference	SE	t	p_{tukey}	$p_{scheffe}$	p_{bonf}	p_{holm}
200, Coh 02%	800, Coh 02%	-2.217	1.620	-1.369	0.998	1.000	1.000	1.000
200, Coh 04%	800, Coh 04%	-8.620	1.620	-5.323	< .001	0.090	< .001	< .001
200, Coh 06%	800, Coh 06%	-11.449	1.620	-7.069	< .001	< .001	< .001	< .001
200, Coh 08%	800, Coh 08%	-13.773	1.620	-8.504	< .001	< .001	< .001	< .001
200, Coh 10%	800, Coh 10%	-13.455	1.620	-8.308	< .001	< .001	< .001	< .001
200, Coh 15%	800, Coh 15%	-11.618	1.620	-7.173	< .001	< .001	< .001	< .001
200, Coh 20%	800, Coh 20%	-11.093	1.620	-6.850	< .001	< .001	< .001	< .001
200, Coh 25%	800, Coh 25%	-8.429	1.620	-5.204	< .001	0.117	< .001	< .001
200, Coh 30%	800, Coh 30%	-7.658	1.620	-4.729	< .001	0.279	< .001	< .001
200, Coh 50%	800, Coh 50%	-1.480	1.620	-0.914	1.000	1.000	1.000	1.000
Note. **P-value adjusted for comparing a family of 190**								
Note. **Results are averaged over the levels of: order, Platform**								

Table S2. Post Hoc Comparisons - Duration ✻ Coherence.
